# The Interaction of Mandarin Fish DDX41 with STING Evokes type I Interferon Responses Inhibiting Ranavirus Replication

**DOI:** 10.3390/v15010058

**Published:** 2022-12-24

**Authors:** Xiao-Wei Qin, Zhi-Yong Luo, Wei-Qiang Pan, Jian He, Zhi-Min Li, Yang Yu, Chang Liu, Shao-Ping Weng, Jian-Guo He, Chang-Jun Guo

**Affiliations:** 1 State Key Laboratory for Biocontrol & Southern Marine Science and Engineering Guangdong Laboratory (Zhuhai), School of Marine Sciences, Sun Yat-sen University, 135 Xingang Road West, Guangzhou 510275, China; 2 Guangdong Province Key Laboratory for Aquatic Economic Animals, and Guangdong Provincial Key Laboratory of Marine Resources and Coastal Engineering, Sun Yat-sen University, 135 Xingang Road West, Guangzhou 510275, China

**Keywords:** DDX41, STING, ranavirus, interferon, innate immune, mandarin fish, MRV

## Abstract

DDX41 is an intracellular DNA sensor that evokes type I interferon (IFN-I) production via the adaptor stimulator of interferon gene (STING), triggering innate immune responses against viral infection. However, the regulatory mechanism of the DDX41-STING pathway in teleost fish remains unclear. The mandarin fish (*Siniperca chuatsi*) is a cultured freshwater fish species that is popular in China because of its high market value. With the development of a high-density cultural mode in mandarin fish, viral diseases have increased and seriously restricted the development of aquaculture, such as ranavirus and rhabdovirus. Herein, the role of mandarin fish DDX41 (*sc*DDX41) and its DEAD and HELIC domains in the antiviral innate immune response were investigated. The level of *sc*DDX41 expression was up-regulated following treatment with poly(dA:dT) or Mandarin fish ranavirus (MRV), suggesting that *sc*DDX41 might be involved in fish innate immunity. The overexpression of *sc*DDX41 significantly increased the expression levels of IFN-I, ISGs, and pro-inflammatory cytokine genes. Co-immunoprecipitation and pull-down assays showed that the DEAD domain of *sc*DDX41 recognized the IFN stimulatory DNA and interacted with STING to activate IFN-I signaling pathway. Interestingly, the HELIC domain of *sc*DDX41 could directly interact with the N-terminal of STING to induce the expression levels of *IFN-I* and *ISGs* genes. Furthermore, the *sc*DDX41 could enhance the *sc*STING-induced IFN-I immune response and significantly inhibit MRV replication. Our work would be beneficial to understand the roles of teleost fish DDX41 in the antiviral innate immune response.

## 1. Introduction

Asp-Glu-Ala-Asp (DEAD) box 41 (DDX41) is a crucial cytosolic DNA sensor and plays a crucial role in antiviral innate immune response via the adaptor stimulator of interferon gene (STING) to induce type I interferon (IFN-I) response. DDX41 consists of two conserved RecA-like domains (DEAD and HELIC domains), which contain various functional motifs involved in nucleic acid recognition, ATP binding, ATP hydrolysis, and RNA unwinding [1,2,3,4]. In the mammalian DDX41-STING signaling pathway, Bruton’s tyrosine kinase phosphorylates the DDX41 HELIC domain at Tyr414, and the activated DDX41 recognizes double-stranded DNAs (dsDNAs) and bacterial cyclic dinucleotides (CDNs), such as cyclic di-AMP and cyclic di-GMP via its DEAD domain [5,6,7]. DDX41 binds to STING located on the endoplasmic reticulum membrane through its DEAD domain to activate IκB kinase-β and TANK-binding kinase 1 (TBK1); the activated kinases phosphorylate IκB and interferon regulatory factor (IRF) -3/-7 trigger the production of proinflammatory cytokines and IFNs [7,8,9]. The HELIC domain of DDX41 regulates the DEAD domain to bind with dsDNAs and CDN but not interact with STING [3,6]. The teleost fish DDX41-STING pathway also plays an important role in antiviral immunity [10,11,12,13,14]. However, whether fish DDX41 directly recognizes dsDNAs and the roles of the HELIC domain in the STING pathway remains unclear. The HELIC domain of DDX41 might obstruct the DDX41-STING signaling pathway in zebrafish [11], indicating that the HELIC domain regulation of the DDX41-STING pathway in fish might be different from that in mammals.

Mandarin fish (*Siniperca chuatsi*) is a cultured freshwater species that is economically important due to its high market value in China. However, the spread and outbreaks of viral diseases, e.g., Mandarin fish ranavirus (MRV), Infectious spleen and kidney necrosis virus, and Siniperca chuatsi rhabdovirus, have caused remarkable loss to the mandarin fish culture industry [15]. To achieve a better understanding of the antivirus immune responses, several IFN-related cytokines or effectors that exert crucial roles in viral replication have been identified [16]. Mandarin fish STING (*sc*STING) displays critical antiviral activity against fish viruses to activate IFN-I immune response [17].

In this study, mandarin fish DDX41 (*sc*DDX41) regulating the *sc*STING-dependent antiviral pathway was identified, and the roles of *sc*DDX41 and its DEAD and HELIC domains in regulating IFN-I responses were investigated.

## 2. Materials and Methods

### 2.1. Fish, Cells, and Virus

Fifty mandarin fish (body weight of 75-100 g) were purchased from a farm in Guangdong province and maintained at 27 °C in a laboratory recirculating freshwater system to acclimatize for 2 weeks before being used. Six fish were randomly selected for pathogen detection, following which, five fish were selected for tissue expression analysis and ten fish for viral infection. For tissue sampling, fish were anaesthetized with MS-222 (40 mg/L, Sigma-Aldrich, St. Louis, MO, USA). All animal experiments were performed in accordance with the regulation for animal experimentation of Guangdong Province, China, and approved by the Ethics Committee of Sun Yat-sen University (No. 2019121705). The MRV strain NH-1609 (GenBank: MG941005.1) was originally isolated from disease-infected mandarin fish and preserved in our laboratory [18]. The mandarin fish fry (MFF-1) cell line was cultured in Dulbecco’s modified Eagle medium (DMEM; Gibco, Grand Island, NY, USA) supplemented with 10% fetal bovine serum (FBS; Gibco, Grand Island, NY, USA) at 27 °C under a humidified atmosphere containing 5% CO_2_ [19].

### 2.2. Antibodies and Reagents

Mouse monoclonal anti-c-myc antibody, mouse monoclonal anti-flag antibody, poly(I:C), and poly(dA:dT) were obtained from Sigma-Aldrich (St. Louis, MO, USA). Rabbit polyclonal anti-YFP antibody was from Abbkine (California, USA).

### 2.3. Molecular Cloning of scDDX41 cDNA

Total RNAs were extracted from MFF-1 cells, and cDNAs were synthesized using previously described methods [17]. The full-length cDNA sequence of *sc*DDX41 was obtained by rapid amplification of cDNA-end (RACE) reactions using a SMARTer RACE cDNA Amplification Kit (Clontech, Tokyo, Japan) in accordance with the manufacturer’s instruction using the primers listed in Table 1. The PCR products were purified, cloned into the pMD19-T vector (Clontech, Tokyo, Japan) and sequenced (Thermo Fisher Scientific, Waltham, MA, USA).

### 2.4. Sequence Analysis

Homology sequences were obtained using the BLAST program at the National Center for Biotechnology Information (http://www.ncbi.nlm.nih.gov/blast). The deduced amino acid sequence of *sc*DDX41 was analyzed using the Simple Modular Architecture Research Tool (SMART) program (http://smart.emblheidelberg.de/). A phylogenetic tree was constructed using the Bootstrap Neighbor-Joining method of the Molecular Evolutionary Genetics Analysis (MEGA) software v10.0 program. Bootstrap sampling was reiterated 1000 times.

### 2.5. Tissue Expression Profiles of scDDX41

Total RNAs were extracted from the gill, fin, spleen, intestine, brain, head kidney, hind kidney, middle kidney, blood, fat, heart, liver, and muscles using the SV Total RNA Isolation Kit (Promega, Madison, Wisconsin, USA) in accordance with manufacturer’s instructions to detect the tissue distribution pattern of *sc*DDX41 in healthy fish. The expression of *sc*DDX41 in various tissues was determined by quantitative reverse-transcriptase PCR (RT-qPCR) using the primers in Table 2.

### 2.6. Plasmid Construction and Mutations

The full-length and various domain-deleted mutants of *sc*DDX41 cDNA fragments were amplified by PCR using specific primers (Table 1) and then inserted into the pCMV-myc (Clontech, Tokyo, Japan) or pEYFP-N1 vector (Clontech, Tokyo, Japan) to construct eukaryotic expression vectors including pCMV-myc-*sc*DDX41 (full-length), pCMV-myc-*sc*DEAD (181–413 amino acids, aas), pCMV-myc-*sc*HELIC (428–529 aas), and pEYFP-*sc*HELIC-N1 (428–529 aas). All plasmids were confirmed by sequencing.

### 2.7. Dual-Luciferase Reporter Assays

Cells were cultured in 24-well plates for 24 h and co-transfected with the IFN-β-luc, or NF-κB-Luc plasmid (0.4 μg), tested plasmid (0.4 μg), and pRL-TK (40 ng) plasmid. After 2 h, the transfection mixture was replaced with 500 µL of DMEM with 10% FBS. After 36 h of transfection, cells were harvested and lysed in 200 µL of Passive Lysis Buffer (Promega, Madison, Wisconsin, USA). Total cell lysates were subjected to a Dual-Luciferase Reporter Gene Assay Kit (Promega, Madison, Wisconsin, USA) in accordance with the manufacturer’s instructions. Luciferase activities were measured using Glomax (Promega, Madison, Wisconsin, USA). Experiments were performed in triplicate, and data represent the mean of at least three independent experiments. All experiments were performed in at least three independent trials with three technical replicates.

### 2.8. RT-qPCR

The RT-qPCR reactions were performed with a SYBR premix ExTaqTM (Takara, Tokyo, Japan) on a LightCycler^®^ 480 instrument (Roche Diagnostics, Switzerland) as previously described [20]. The primers for RT-qPCR were designed using Primer premier software v5.0 program (Applied Biosystems, Table 2). The expression of each transcript was normalized to the expression of the *β-actin* gene. All data of the target genes were analyzed using the Q-gene statistics add-in, followed by an unpaired sample *t*-test. Statistical significance was accepted at *p*<0.05, and high significance was accepted at *p*<0.01. All data are expressed as mean ± standard deviation (SD).

### 2.9. Absolute Quantitative Real-time PCR (qPCR) and TaqMan Probe

Virus load was determined by viral genomic copies using qPCR. DNA was extracted using the DNA isolation Mini Kit (Vazyme, Nanjing, China). The levels of viral genomic copies were quantified by the amount of viral major capsid protein (*mcp*) gene copies, which were determined with the method of standard curve. Specific primers and probes were designed from the partial MRV *mcp* sequence of NH-1609 using Primer Express v3.0 program. The forward primer 5′-GCCAAAAACCTTGTCCTTCCT -3′, reverse primer 5′-TCGTTGTAAGGCAGGGTGACT-3′, and the probe 5ʹ-FAM-CCCTTCTTTTTCGGCAGAGA-BHQ1-3ʹ were identified. Each PCR mixture in 10 µL contained 5 µL 2× Real PCR Easy TM Mix-Taqman (FOREGENE, Chengdu, China), 400 nM primer each, 200 nM probe, and 100 ng total DNA template. The program started with an initial denaturation at 94 °C for 3 min, followed by 40 cycles at 94 °C for 10 s and 60 °C for 30 s. The amplification and date analysis were carried out in Roche LightCycler^®^ 480 (Roche Diagnostics, Switzerland).

### 2.10. Determinations of Virus Titer

Cells were seeded into 96-well dishes. After growing overnight, cell density exceeded 80%. Briefly, cells cultivated in 96-well plates were infected with serial 10-fold dilutions of MRV-containing samples, with eight wells per dilution, and 100 μL of culture medium without virus was placed in eight wells as negative control. Then, the 96-well dishes were incubated at 27 °C. After 7 days of incubation, the number of positive and negative wells were recorded; the TCID_50_ was calculated using the Spearman–Karber method as previously described [20].

### 2.11. Co-Immunoprecipitation (Co-IP) and Western Blot (WB) Analysis

The interaction between *sc*DDX41 and *sc*STING was analyzed via Co-IP assay. Cells seeded on 60 mm dishes (1 × 10^7^ cells/dish) were transfected with 8 μg of empty plasmid or various expression plasmids including pCMV-myc-*sc*DDX41 plus pCMV-flag-*sc*STING (at a ratio of 1:1), pCMV-myc-*sc*DDX41 plus pCMV-flag (at a ratio of 1:1), and pCMV-myc plus pCMV-flag-*sc*STING (at a ratio of 1:1). At 24 h post-transfection, the cells were placed in an ice-cold cell lysis buffer (Beyotime, Shanghai, China) containing a cocktail protease inhibitor (Merck Millipore, Billerica, Massachusetts, USA) for 30 min at 4 °C. The cell lysates were centrifuged at 12,000 g for 15 min at 4 °C, precipitated with mouse anti-myc mAb or mouse anti-flag mAb at 37 °C for 60 min, and incubated with 50 µL of protein G-magnetic beads (Thermo Fisher Scientific, Waltham, MA, USA) for 60 min. The beads were washed four times with lysis buffer and eluted with an SDS loading buffer by boiling for 10 min. The transfected cells were harvested at 36 h post-transfection and lysed for WB assay as previously described [17]. Protein bands were visualized using a High-sig chemiluminescence Western blot substrate kit (Tanon, Shanghai, China).

### 2.12. Electrophoretic Mobility Shift Assay (EMSA)

Cells were transfected with pCMV-myc-*sc*DDX41, pCMV-myc-GFP, or pCMV-myc. After 24 h, the cells were collected, and nuclear proteins were extracted using NE-PER Nuclear and Cytoplasmic Extraction Reagents (Thermo Fisher Scientific, Waltham, MA, USA). The 5′ biotin-labeled IFN stimulatory DNA (ISD) probe (5′-TACAGATCTACTAGTGATCTATGACTGATCTGTACATGATCTACA-3′) was synthesized by Thermo Fisher Scientific (Waltham, MA, USA). In the competitive binding experiment, the unbiotinylated probes at 10- or 100-fold molar excess over the labeled probes were used to challenge the complexes of wild-type probes and proteins. EMSA was performed using a LightShift Chemiluminescent EMSA kit (Thermo Fisher Scientific, Waltham, MA, USA). The nuclear proteins (10 μg) were incubated with 20 fmol probes for binding reactions between the probes and proteins, separated by 5% native PAGE, transferred to positively charged nylon membranes (Roche Diagnostics, Switzerland), and cross-linked by UV light. The biotin-labeled DNA on the membrane was detected by chemiluminescence and developed on X-ray films as mentioned earlier [21].

### 2.13. Pull-Down Assay

The 5′ biotinylated sense strand of ISD was mixed with its complementary unlabeled DNA and annealed to make double-stranded bio-ISD. For negative controls and competition assays, unlabeled ISD and Bio-ISD, each at a final concentration of 1 μM, were incubated with Dynabeads M-280 Streptavidin (Thermo Fisher Scientific, Waltham, MA, USA) for 30 min at 4 °C in binding buffer (50 mM Tris-HCl, pH 7.5, 150 mM NaCl, 10% glycerol, 0.5 mM EDTA, 1 mM 2-mercaptoethanol, and 0.5% Nonidet P-40). The beads were washed with binding buffer three times and incubated with the proteins for 1 h at 4 °C in binding buffer. The beads were washed with binding buffer four times. The bound proteins were eluted by boiling for 10 min at 100 °C using an SDS-PAGE sample buffer [6].

## 3. Results

### 3.1. Molecular Characteristics of scDDX41

**Figure 1:**
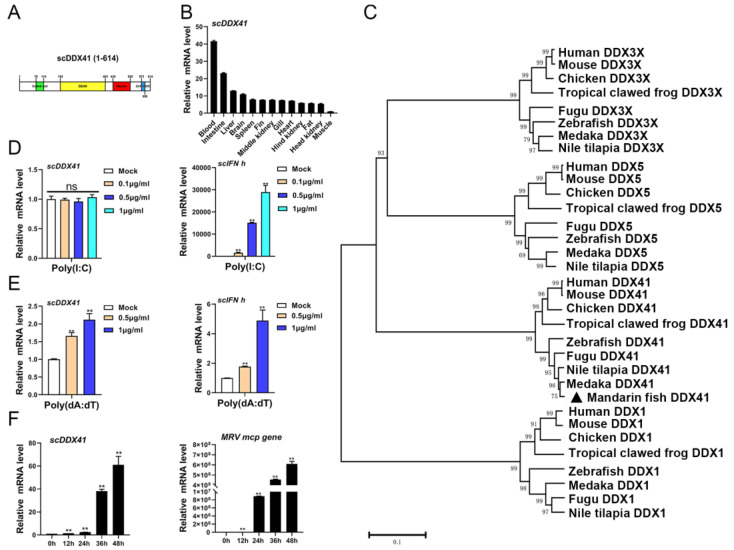
(**A**) SMART analysis of DDX41s. Coiled coil domain, DEAD domain, HELIC domain, and ZnF_C2HC domain were labeled in the sequence. (**B**) Distributions of *sc*DDX41 expression in different tissues of mandarin fish. The expression levels of scDDX41 were detected by RT-qPCR. (**C**) Phylogenetic tree of DDX41 proteins from various species. A phylogenetic tree was constructed using the Neighbor-Joining method in MEGA v10.0, with 1000 bootstrap replications. The bootstrap values were indicated at the nodes of the tree. (**D**) Expression levels of *scDDX41* in cells treated with poly(I:C) at indicated times. (**E**) Expression levels of *scDDX41* in cells treated with poly (dA:dT) at indicated times. (**F**) Expression levels of *scDDX41* in cells infected with MRV at indicated times. The *β-actin* gene served as internal control to calibrate the cDNA template for all samples. Vertical bars represent ±SD (*n* = 3). Statistical significance was indicated by asterisks, with ** referring to *p* < 0.01. ns, non-significant.

The cDNA fragment of *sc*DDX41 was obtained from transcriptome data (unpublished). The RACE method was used to clone the full-length cDNA of *sc*DDX41. The full-length cDNA of *sc*DDX41 (MN482034) is 2250 bp and includes a 5′-untranslated region (UTR) of 133 bp, a 3′-terminal UTR of 272 bp, and an 1845 bp open reading frame encoding a protein of 614 aas. SMART analysis and amino acid alignment showed that *sc*DDX41 contains a coiled coil region (78–114 aas), a DEAD domain (192–403 aas), a helicase superfamily C-terminal (HELIC) domain (438–519 aas), and a ZnF_C2HC domain (573–589 aas) (Figure 1A). The expression levels of *sc*DDX41 in various tissues were examined by RT-qPCR. The transcriptions levels of *sc*DDX41 were constitutively detected in all the selected tissues (including gill, hind kidney, fin, blood, spleen, middle kidney, head kidney, intestine, fat, liver, heart, brain, and muscles) under healthy conditions. The expression of *sc*DDX41 in blood was higher than those in the other tissues and was approximately 42 times that in the muscles (Figure 1B). Phylogenetic analysis showed that *sc*DDX41 was clustered with DDX41 proteins from other fish to form an exclusive group and was merged with mammalian DDX41 proteins into a large group with high bootstrap probability, which differed from the other DDX41 family members, including DDX1, DDX3X, and DDX5 (Figure 1C).

### 3.2. Expressions of scDDX41 in Response to Immune Stimulations

The expression profiles of *scDDX41* in response to immune stimulation were evaluated. Cells were treated with double-stranded RNA (dsRNA) analogue poly(I:C), DNA analogue poly(dA:dT) or MRV, and then the transcription levels of *scDDX41* were detected at indicated time by RT-qPCR. The levels of *scDDX41* transcription were not changed by poly(I:C) stimulation at concentrations of 0.1–1 μg/mL (Figure 1D), whereas the levels were significantly increased by poly(dA:dT) stimulation at concentrations of 0.5–1 μg/mL (Figure 1E) or upon infection with MRV (Figure 1F). The levels of type I IFN (*IFN-h*) transcription in the controls were significantly increased by poly(I:C) and poly(dA:dT) stimulations (Figure 1D,E). These observations suggested poly(dA:dT) stimulation or infection with MRV induced the expression of *scDDX41*, indicating that *sc*DDX41 might be involved in innate immune responses.

### 3.3. ScDDX41 and Its Domains Evoked IFN-I and Inflammatory Response

**Figure 2:**
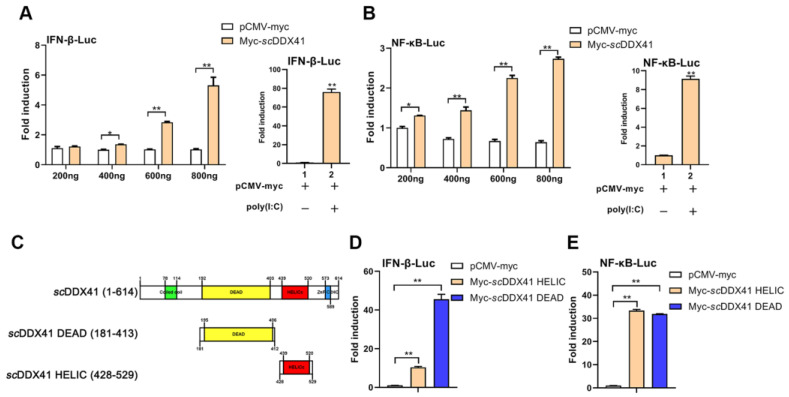
*sc*DDX41 induced the activities of IFN-β-luc and NF-κB-luc promoters. (**A**) Cells were transfected with 0.2, 0.4, 0.6, or 0.8 μg of *sc*DDX41 expression plasmid or an empty vector together with IFN-β-luc (0.4 μg/well) and pRL-TK (0.04 μg/well) plasmids. Luciferase assays were performed 36 h after the transfection. (**B**) Cells were transfected with 0.2, 0.4, 0.6, or 0.8 μg of *sc*DDX41 expression plasmid or an empty vector together with NF-κB-luc (0.4 μg/well) and pRL-TK (0.05 μg/well). Luciferase assays were performed 36 h after the transfection. (**C**) Schematic of full-length and *sc*DDX41 mutants with the DEAD domain, HELIC domain, and residue numbers as indicated. Various *sc*DDX41 fragments were inserted into the C-terminus of pCMV-myc. (**D**,**E**) DEAD and HELIC domains of *sc*DDX41 for IFN and NF-κB activation. Cells were transfected with 0.4 μg/well of various expression plasmids of *sc*DDX41, *sc*DDX41 mutants, or empty vector together with the reporter plasmid 0.04 μg/well pRL-TK as well as 0.4 μg/well of IFN-β-luc or NF-κB-luc plasmid. Luciferase assays were performed 36 h after the transfection. All luciferase assays were repeated at least three times, and data are means ±SD (*n* = 3) from single representative experiments. * *p* < 0.05, ** *p* < 0.01 between normal cells and stimulated cells.

To investigate the roles of *sc*DDX41 in innate immune response, the IFN-I and NF-κB signaling pathway were detected. As shown in Figure 2A,B, overexpression of *sc*DDX41 induced the activities of IFN-β-luc and NF-κB-luc in a dose-dependent manner, compared with the overexpression of pCMV-myc (negative control). The activities were also significantly increased in cells treated with poly (I:C), which was set as positive control. These results suggested that *sc*DDX41 was capable of activating IFN-β and NF-κB promoters. According to SMART analysis, *sc*DDX41 possessed two important domains, namely, N-terminal DEAD domain (181–413 aas) and a C-terminal HELIC domain (428–529 aas) (Figure 2C). To investigate the roles of DEAD and HELIC domains in the IFN-I and NF-κB signaling pathway, various mutants of *sc*DDX41 were constructed. As shown in Figure 2D,E, the activities of IFN-β-luc and NF-κB-luc were significantly increased by the DEAD domain compared with those in the full length of *sc*DDX41, which was similar to previous studies [6,14]. Interestingly, the HELIC domain could strongly induce the activities of IFN-β-luc and NF-κB-luc.

**Figure 3:**
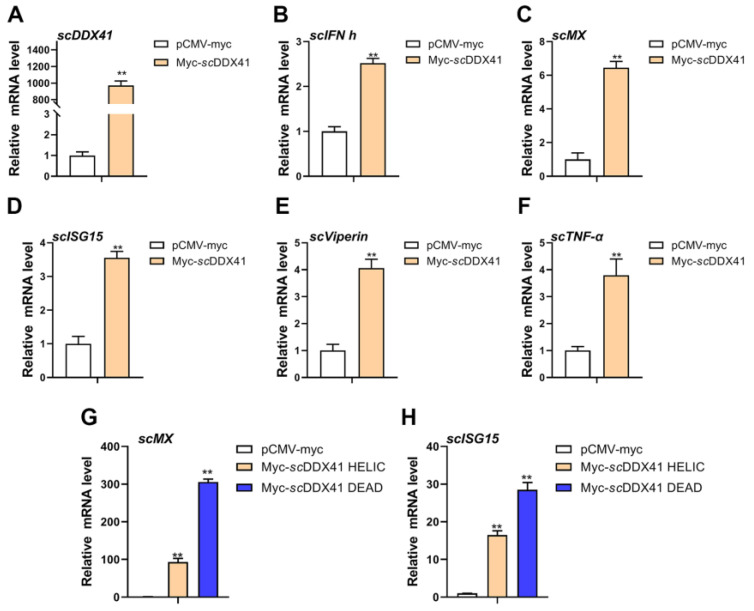
Overexpression of *sc*DDX41 induces the expression of IFN-I, ISGs, and inflammatory cytokines. After transfection with *sc*DDX41-myc or pCMV-myc at 24 h, cells were harvested and the expression levels of *scDDX41* (**A**), *scIFN-h* (**B**), *scMx* (**C**), *scISG15* (**D**), *scViperin* (**E**) and *scTNF-α* (**F**) genes were detected. Overexpression of *sc*HELIC and *sc*DEAD induced the expression of *scMx* (**G**) and *scISG15* (H) in MFF-1 cells. The *β-actin* gene served as the internal control to calibrate the cDNA template for all samples. Vertical bars represent ±SD (*n* = 3). Statistical significance is indicated by asterisks, with ** referring to *p* < 0.01.

To further elucidate the roles of *sc*DDX41 and its domains in innate immune responses, the effects on the expressions of innate immune-related genes were investigated using RT-qPCR. As shown in Figure 3, the expression levels of IFN-I signaling-related genes (*scIFN-h*, *scMx*, *scISG15*, and *scViperin* genes; Figure 3B–E) and pro-inflammatory factor (*sc*TNF-α gene; Figure 3F) in the cells transfected with *sc*DDX41-myc were significantly higher than those in the cells transfected with the control vector (pCMV-myc plasmid) at 24 h post-transfection (*p*<0.01). These observations suggested that *sc*DDX41 evoked IFN and inflammatory responses. Moreover, the DEAD and HELIC domains strongly enhanced the expression levels of *scMx* and *scISG15* compared with the cells transfected with control and *sc*DDX41 plasmids (Figure 3G,H), suggesting that the DEAD and HELIC domains of *sc*DDX41 were important in IFN-I immune responses.

### 3.4. ScDDX41 Interacted with scSTING

**Figure 4:**
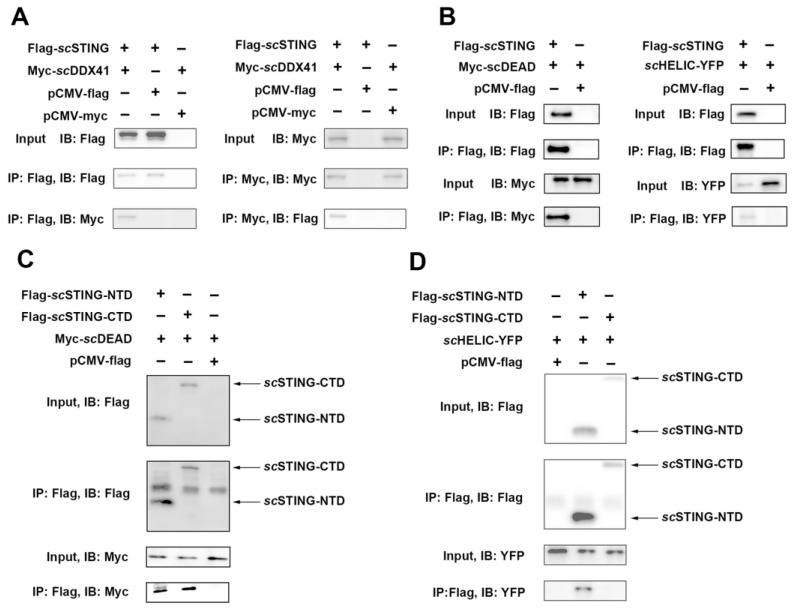
Identification of the interaction between *sc*DDX41 and *sc*STING by Co-IP assay. (**A**) *sc*DDX41 interacted with *sc*STING. Cells were transfected with the indicated plasmids. At 36 h post-transfection, the cell lysates were precipitated with an anti-flag or anti-myc mAb in conjunction with protein G-Sepharose beads and detected by WB analysis using anti-myc or anti-flag mAbs. The expression of the transfected proteins was analyzed by immunoblotting with anti-myc and anti-flag mAbs. (**B**) DEAD and HELIC domains of *sc*DDX41 interacted with *sc*STING. Cells were co-transfected with DEAD-myc, HELIC-YFP, and *sc*STING-flag or empty vector. Immunoprecipitation assays with anti-flag antibody (IP: Flag) and Western blot analysis were performed with anti-Flag, anti-myc, or anti-YFP antibodies. (**C**) DEAD domain interacted with *sc*STING-NTD and *sc*STING-CTD. Cells were co-transfected with myc-DEAD and flag-*sc*STING-NTD, flag-*sc*STING-CTD, or empty vector. Immunoprecipitation assays with anti-flag antibody (IP: Flag) and WB analysis were performed with anti-Flag or anti-myc antibodies. (**D**) HELIC domain interacted with *sc*STING-NTD but not with *sc*STING-CTD. Cells were co-transfected with HELIC-YFP and flag-*sc*STING-NTD, flag-*sc*STING-CTD, or empty vector. Immunoprecipitation assays with anti-flag antibody (IP: Flag) and WB analysis were performed with anti-flag or anti-YFP antibodies.

To investigate whether the function of *sc*DDX41 in IFN-I expression depends on *sc*STING, *sc*DDX41 and its domain interactions with *sc*STING were determined by Co-IP assay. The results showed that anti-flag mAb precipitated myc-tagged-*sc*DDX41 in cells co-expressed with flag-tagged-*sc*STING and anti-myc mAb precipitated flag-tagged-*sc*STING in cells co-expressed with myc-tagged-*sc*DDX41 (Figure 4A). Co-IP experiments were conducted on the interaction between DEAD and HELIC with *sc*STING, respectively. Importantly, in contrast to mammalian DDX41, the HELIC domain of *sc*DDX41 also directly interacted with *sc*STING (Figure 4B) [3,6]. Furthermore, the interactions of the *sc*STING domain, *sc*STING N-terminal domain (NTD), and *sc*STING C-terminal domain (CTD) with the DEAD and HELIC domain of *sc*DDX41 were explored. As shown in Figure 4C,D, the DEAD domain of *sc*DDX41 was bound to both *sc*STING NTD and CTD, whereas the HELIC domain of *sc*DDX41 was bound to *sc*STING NTD but not to CTD.

**Figure 5:**
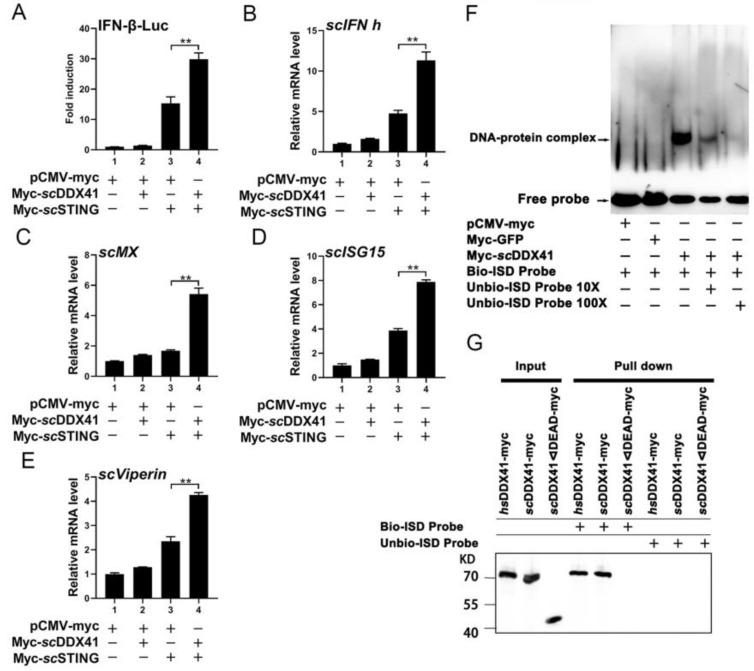
Involvement of *sc*DDX41 in *sc*STING-mediated IFN expression, and *sc*DDX41 recognizes dsDNA through the DEAD domain. (**A**) Cells transfected with an IFN-β-luc 400 ng/well and TK (40 ng/well) plus 400 ng/well) of the expression vectors for pCMV-myc, pCMV-myc and *sc*DDX41-myc, pCMV-myc and *sc*STING-myc, *sc*DDX41-myc, and *sc*STING-myc. Vertical bars represent ±SD (n = 3). Statistical significance is indicated by asterisks, with ** referring to *p* < 0.01. *sc*DDX41 enhanced the IFN-I response induced by STING. (**B**–**E**) Cells seeded in 6-well plates were transfected or co-transfected with *sc*DDX41 (400 ng/well), and *sc*STING (400 ng/well). The cells transfected with pCMV-myc acted as negative control. The expression levels of interferon signaling molecules including *scIFN-h*, *scMx*, *scISG15*, and *scViperin* were examined using RT-qPCR. The *β-actin* gene served as the internal control to calibrate the cDNA template for all samples. Vertical bars represent ±SD (*n* = 3). Statistical significance is indicated by asterisks, with ** referring to *p* < 0.01. (**F**) *sc*DDX41 recognizes ISD through the DEAD domain using EMSA with biotin-labeled (Bio-) or unlabeled (Unbio-) probes ISD. The black lines indicate where parts of the image were joined. (**G**) Immunoblot analysis of the immunoprecipitated purified myc-tagged *hs*DDX41, *sc*DDX41, or *sc*DDX41ΔDEAD recombinant proteins incubated individually with biotinylated ISD and probed with anti-myc antibodies.

To further investigate whether *sc*DDX41 could enhance *sc*STING-induced IFN-I immune response, the expression levels of downstream genes in the cells co-transfected with *sc*DDX41 and *sc*STING were examined. As shown in Figure 5A–E, *sc*DDX41 significantly enhanced the activities of IFN-β-luc and the expression levels of *scIFN-h*, *scMx*, *scISG15*, and *scViperin* genes in the cells co-transfected with *sc*STING. These results suggested that *sc*DDX41 enhanced the *sc*STING-induced IFN-I immune response.

### 3.5. ScDDX41 Recognizes dsDNA through the DEAD Domain

To verify whether teleost fish DDX41 could recognize dsDNAs, the interaction between *sc*DDX1 and interferon stimulatory DNA (ISD) was evaluated using EMSA. As shown in Figure 5F, *sc*DDX41 could bind to the labeled probe of ISD to form a retarded band, whereas the retarded bands of the DNA–protein complex were eliminated when 10× and 100× unlabeled probes were added to competitively bind to the *sc*DDX41 protein. By contrast, no retarded band of the DNA–protein complex was observed in the analysis of the control group performed using nuclear proteins extracted from cells transfected with pCMV-myc-GFP or pCMV-myc empty plasmid. These findings confirmed that *sc*DDX41 could bind to ISD. To further determine whether the DEAD domains of *sc*DDX41 could directly bind to dsDNA, the dsDNA-binding abilities of myc-tagged *sc*DDX41 and myc-tagged *sc*DDX41ΔDEAD were assessed by pull-down assays using 5ʹ-biotinylated ISD (bio-ISD). The myc-tagged human DDX41 (*hs*DDX41) was used as positive control. As shown in Figure 5G, avidin beads plus bio-ISD immunoprecipitated *hs*DDX41 and *sc*DDX41 but not *sc*DDX41ΔDEAD due to the absence of the DEAD domain. Hence, *sc*DDX41 could bind to DNA through the DEAD domain. These results indicated that *sc*DDX41 recognized dsDNA through the DEAD domain.

### 3.6. ScDDX41 Inhibits the Replication of Ranavirus

**Figure 6:**
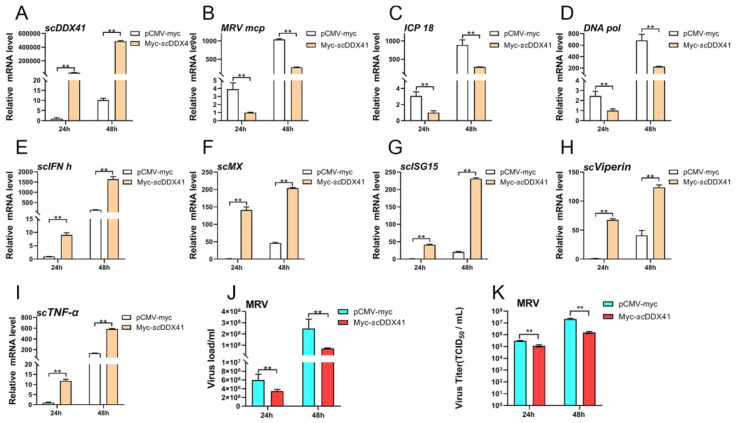
Overexpression of *sc*DDX41 attenuates MRV infection. After transfection with *sc*DDX41-myc or pCMV-myc at 24 h, cells were infected with MRV and harvested for RT-qPCR independently at the indicated time points. (**A**) Expression levels of *sc*DDX41 in cells infected with MRV at indicated times. (**B**–**D**) Expression levels of *mcp*, *ICP-18*, and *DNA pol* genes in cells infected with MRV at indicated times. (**E**–**I**) Expression levels of *scIFN-h*, *scMx*, *scISG15*, *scViperin*, and *scTNF-α* genes in cells infected with MRV at indicated times. (**J**) Correlation of viral load in samples measured by TaqMan qPCR. (**K**) The titer of virus infection was measured on a 96-well cell culture plate via the finite dilution method. *β-actin* gene served as the internal control to calibrate the cDNA template for all samples. Vertical bars represent ±SD (*n* = 3). Statistical significance is indicated by asterisks, with ** referring to *p* < 0.01.

To investigate the potential antiviral roles of *sc*DDX41, the transcription level of MRV viral *major capsid protein* (*mcp*), *ICP-18* and *DNA polymerase* (*DNA pol*) genes were evaluated in *sc*DDX41-overexpressing cells. As shown in Figure 6A–D, the transcriptions of MRV *mcp*, *ICP-18*, and *DNA pol* genes were significantly inhibited in *sc*DDX41-overexpressing cells (A) compared with that in the cells transfected with the control vector at 24 and 48 h post-infection (*p* < 0.01). The relative expression levels of *scIFN-h* (E), *scMx* (F), *scISG15* (G), *scViperin* (H), and *scTNF-α* (I) genes in the cells transfected with *sc*DDX41-myc were significantly higher than those in the cells transfected with pCMV-myc at 24 and 48 h (*p* < 0.01) post-MRV infection. Moreover, the level of virus load and virus titers (TCID_50_) was also suppressed by *sc*DDX41 at 24 and 48 h post-infection, compared to those in the control group (Figure 6J,K). These findings suggested that *sc*DDX41 exerted antiviral activity.

## 4. Discussion

DDX41, a member of the DExD/H-box helicase superfamily, is involved in intracellular DNA recognition and triggers innate immune responses against viral infection [3,6,11]. Current knowledge about DDX41 mainly originates from humans and mice [6]. To fully understand the biological function of DDX41 in innate immunity, particularly of lower vertebrates such as teleost fish, scholars have cloned and studied DDX41 from olive flounder (*Paralichthys olivaceus*), zebrafish (*Danio rerio*), orange spotted grouper (*Epinephelus coioides*), Nile tilapia (*Oreochromis niloticus*), and grass carp (*Ctenopharyngodon idella*) for the past few years [10,11,12,13,14]. *P. olivaceus* DDX41 directly sensed cyclic di-GMP and triggered an IFN immune response to enhance the antiviral effect [21]. *D. rerio* DDX41 activated NF-κB and IFN-I signaling pathways through the interaction of the DEAD domain with STING [11]. *E. coioides* DDX41 enhanced MAVS- and TBK1-induced IFN and inflammatory immune responses [12]. *C. idella* DDX41 interacted with STING to induce the phosphorylation and nuclear translocation of IRF7 and then initiated *IFN-I* and *ISG15* expressions [13]. However, no direct evidence is available to support the interaction of DDX41 with DNA (dsDNA, CDNs, or ISD) in teleost fish. In the present study, the DEAD domain of *sc*DDX41 is a key region for binding to DNA (ISD) and interaction with STING to trigger IFN and inflammatory immune responses. Hence, *sc*DDX41 is an important DNA pattern-recognition receptor for STING-dependent type I IFN responses in teleost fish.

DDX41 can directly bind to the STING-N-segment transmembrane region through the DEAD domain and subsequently promotes the activation of TBK1 and IRF3, leading to IFN-I production; meanwhile, the HELIC domain of DDX41 regulates the binding ability of the DEAD domain to dsDNA but does not interact with STING [6,22]. Teleost fish DDX41 also interacts with STING via the DEAD domain; however, the HELIC domain of *D. rerio* DDX41 might obstruct the DDX41-STING signaling pathways [11]. Although the DDX41 of fish is highly homologous with the DDX41 from other species, there are differences between the STING of fish and those of other species [17,23]. The N-terminal TM domain of fish STING presents diversity; for example, grass carp STING has three TM structure domains [22], while grouper STING contains four TM domains [24], and the *sc*STING contains five TM domains [17]. The TM domains of STING in fishes may lead to different mechanisms from those of mammals due to their high degree of unconserved sequences. The signal activation of STING depends on the IRF3 and NFkB. Different from the mammalian STING, the zebrafish STING can significantly stimulate a downstream NF-κB signal but the IRF3-IFN signal is weaker, which is due to the difference of the C terminal of STING [23,25]. Those findings indicated the difference in the activation of the DDX41-STING pathway between mammals and teleost fish. Interestingly, the DEAD domain of *sc*DDX41 could directly interact with not only *sc*STING-NTD but also *sc*STING-CTD, and the HELIC domain of *sc*DDX41 could also directly interact with *sc*STING-NTD. Hence, the DEAD and HELIC domains of *sc*DDX41 contributed to the interaction of *sc*DDX41 with *sc*STING to induce STING-dependent IFN-I and inflammatory immune responses. As such, the activation of the DDX41-STING pathway might differ between mammals and teleost fish. The detailed mechanisms of the DDX41 HELIC domains in synergistically regulating the STING-mediated innate immune response in fish should be further studied.

In summary, our study demonstrated that the *sc*DDX41 activated the IFN-I responses by interacting with *sc*STING though the DEAD and HELIC domain, and the *sc*DDX41-mediated STING pathway possessed antiviral activity against MRV infection. This work will help us to understand the roles of teleost fish DDX41 and its HELIC domain in innate immune responses.

## Figures and Tables

**Table 1:** Primers used for cloning.

Names	Sequence (5′–3′)
5′ RACE-F	CTAATAGCACTCACTATAGGGCAAGCAGTGGTATCAACGCAGAGT
5′ RACE-R	TGAGATGCTGATGCTGGTCAAGGAG
3′ RACE-F	TGATGGATCTTAAAGCCCTGC
3′ RACE-R	ACTCTGCGTTGATACCACTGCTTGCCCTATAGTGAGTGCTATTAG
*sc*DDX41-F	CGGAATTCCGGAGACCGACAATCGACCC
*sc*DDX41-R	GGGGTACCTTAGAAGTCCATTGAGCTATGAGC
*sc*DEAD-F	CGGAATTCCGCCACCAGCAATTCTAAAAGG
*sc*DEAD-R	GGGGTACCTTACATCTTGGCCTCCTCTTTG
*sc*HELIC-F	CGGAATTCCGCTTTTATTTGCTGAGAAGAAGG
*sc*HELIC-R	GGGGTACCTTAATTAATGAACGTAGTGGC
*sc*HELIC-YFP-F	CGGAATTCCGATGCTTTTATTTGCTGAGAAGAAG
*sc*HELIC-YFP-R	GGGGTACCCCATTAATGAACGTAGTGGC
ISD-F	TACAGATCTACTAGTGATCTATGACTGATCTGTACATGATCTACA
ISD-R	TGTAGATCATGTACAGATCAGTCATAGATCACTAGTAGATCTGTA

**Table 2:** Primers used for RT-qPCR.

Gene Names	Primers	Sequences (5′–3′)	Primer Efficiency
*scDDX41*	Forward Reverse	ACGATTATGTTCCGTACATTCCAGTCAA TCCTCATCCCTCTGCTCCTCCC	0.99
*MRV mcp*	Forward Reverse	GTCACCCTGCCTTACAACGAAA CACGATGGGCTTGACTTCTCC	0.96
*MRV ICP-18*	Forward Reverse	AGTTTGACGCCAGCTTTCACG TGCCATACCGTCGCACTCG	0.98
*MRV DNA polymerase*	Forward Reverse	GGCGTCAAGTGCCATCAG CAGGCGAGACAGTCTTCCAAT	0.98
*IFN-h*	Forward Reverse	CGCTCTGCTGTGATTGGC GGGACTCCACCTCTGCCTTT	0.99
*scMX*	Forward Reverse	GGATTCTGACATCGGGAGCAA GTGCAGTAGACTCATGCTGT	0.98
*scISG15*	Forward Reverse	CGACGAGACTGTGAGCGACTTC CATTCATCATCTCCCTGCCTTGGT	0.99
*scViperin*	Forward Reverse	CCAAGAGGGGCCTCAAACTT CTGACACTTGGGAGCTGGAG	0.95
*scTNF-α*	Forward Reverse	AGCCAGGCATCGTTCAGAGTCT CTGTCCTCCTGAGCGGTGTCTT	0.96
*β-actin*	Forward Reverse	CCCTCTGAACCCCAAAGCCA CAGCCTGGATGGCAACGTACA	0.96

## Data Availability

All data are either provided in the Article and its Supplementary Information or are available from the corresponding author upon request.
